# Effects of Two Weekly Servings of Cod for 16 Weeks in Pregnancy on Maternal Iodine Status and Infant Neurodevelopment: Mommy's Food, a Randomized-Controlled Trial

**DOI:** 10.1089/thy.2020.0115

**Published:** 2021-02-12

**Authors:** Maria Wik Markhus, Mari Hysing, Lisa Kolden Midtbø, Ive Nerhus, Synnøve Næss, Inger Aakre, Ingrid Kvestad, Lisbeth Dahl, Marian Kjellevold

**Affiliations:** ^1^ Seafood, Nutrition and Environmental State, Institute of Marine Research, Bergen, Norway.; ^2^ Regional Centre for Child and Youth Mental Health and Child Welfare, NORCE Norwegian Research Centre, Bergen, Norway.; ^3^ Department of Psychosocial Science, Faculty of Psychology, University of Bergen, Bergen, Norway.

**Keywords:** iodine, thyroid hormones, pregnancy, neurodevelopment, RCT, infants

## Abstract

***Background:*** Mild-to-moderate iodine deficiency is still present in many countries, particularly in pregnant women. Observational studies suggest that mild-to-moderate iodine deficiency during pregnancy may be associated with impaired thyroid function and child neurodevelopment. Randomized-controlled food trials to increase iodine status are scarce. We assessed the impact of an increased intake of cod during pregnancy on maternal iodine status and infant neurodevelopment.

***Methods:*** In this randomized-controlled trial, pregnant women in Bergen, Norway, recruited through Haukeland University Hospital, were randomly assigned (1:1) to an intervention of 200 g of cod twice a week for 16 weeks (gestational week 20–36) or to continue with their standard diet (control group). Randomization was done by lottery. Primary outcome was urinary iodine concentration (UIC) (spot samples from six consecutive days) measured postintervention. Secondary outcome was infant neurodevelopment assessed by the cognitive, language, and motor scales of the *Bayley Scales of Infant and Toddler Developmental* third edition (Bayley-III) at 11 months of age. In addition, maternal thyroid function was measured (thyrotropin [TSH], free triiodothyronine [fT3], free thyroxine [fT4]) at baseline and postintervention.

***Results:*** Between January 2016 until February 2017, 137 women were recruited. Postintervention UIC was higher in the intervention group (*n* = 61) [median (interquartile range, IQR) 98 (64–145) μg/L], compared with control (*n* = 61) [median (IQR) 73 (52–120) μg/L] (*p* = 0.028), also after adjusting for baseline UIC (*p* = 0.048). Infants of mothers in the intervention group had a lower cognitive composite score on the Bayley-III compared with the control group (*p* = 0.045). There were no group differences in the Bayley III language- or motor composite scores. Maternal thyroid hormones (TSH, fT3, fT4) did not differ between the groups postintervention.

***Conclusions:*** Increased cod intake during pregnancy improved the iodine status in women with mild-to-moderate iodine deficiency, however, did not affect thyroid function. The negative effect on cognition should be followed up to assess whether this is a stable effect over time. More studies are warranted to enable good health advice on iodine nutrition in pregnancy. ClinicalTrials.gov NCT02610959. Registered November 20, 2015.

## Introduction

Although substantial progress has been made toward improving iodine status worldwide, iodine deficiency remains a significant health problem in low-, middle-, and high-income countries (1). Severe iodine deficiency is almost completely eradicated, mainly through salt iodization programs (2). However, mild-to-moderate iodine deficiency is still present in many countries, particularly in women of childbearing age, where Europe is the continent with the highest prevalence of iodine deficiency worldwide (3).

Iodine is an essential micronutrient because of its incorporation in the thyroid hormones triiodothyronine (T3) and thyroxine (T4). Pregnancy is a period of increased iodine requirements to maintain euthyroidism due to transfer of iodine and thyroid hormones to the fetus (4). Consequently, pregnant women are vulnerable to iodine deficiency.

The World Health Organization (WHO) recommends an iodine intake of 150 μg/day for nonpregnant women and 250 μg/day for pregnant women and the Nordic Nutrition Recommendations recommend 150 μg/day for nonpregnant women, 175 μg/day for pregnant women, and 200 μg/day for lactating women (2,5). WHO recommends using the median urinary iodine concentration (UIC) to assess iodine status in population groups. A median UIC of 150–250 μg/L indicates optimal iodine nutrition in pregnant women, while a UIC <150 is considered insufficient (2). The Nordic Nutrition Recommendations of 175 μg/day corresponds to a UIC of ∼100 μg/L. Observational studies have found adverse health effects when UIC falls below 100 μg/L, indicating that this may be proposed as a cutoff for sufficiency during pregnancy (6,7).

Norway is currently a country of documented mild-to-moderate iodine deficiency in pregnant women with median UIC ranging from 75 to 92 μg/L (8–11). This may be caused by the decrease in intake of milk and lean fish during the last decade (12). Norway has no iodized salt, and milk, dairy products, and seafood are the main dietary iodine sources of the total iodine intake (8,13). After the completion of this trial, Norwegian authorities have recommended iodine supplementation of 150 μg/day to pregnant women who have a lower daily intake than 6 dL of cow's milk/yogurt (but eat white saltwater fish regularly), or eat little/no white saltwater fish and at the same time have a lower daily intake than 8 dL cow's milk/yogurt.

Severe iodine deficiency is a well-known risk factor for cognitive deficits in children (14,15). The effects of mild-to-moderate iodine deficiency on cognitive development are less certain. Few intervention studies on maternal iodine supplementation on child development exist and randomized control trials (RCTs) have been urgently called for (16,17). To the best of our knowledge, this is the first intervention trial in pregnant women with iodine-rich food (cod) to study its effects on maternal iodine status and infant neurodevelopment. The primary aim of this RCT was to investigate if an increased intake of cod during pregnancy has an effect on maternal iodine status, and secondarily if it has an effect on infant neurodevelopment at 11 months of age.

## Materials and Methods

### Study design and participants

This study was a two-arm RCT with a primary aim of studying the effect of increased cod intake during pregnancy on maternal iodine status, and a secondary aim of determining infant neurodevelopment when the children were 11 months old. Participants were recruited through the Women's Clinic at Haukeland University Hospital, Health region West, Norway. Approximately 5000 women give birth at the Women's Clinic annually. Once a pregnant woman was enrolled, the study investigators made every reasonable effort to follow the participant closely for the entire trial period to ensure the best possible retention. Before all the visits, the participants were reminded about the upcoming appointment.

From January 2016 until February 2017, information regarding the intervention trial was included in the invitation from the Women's Clinic at the time of routine ultrasound in gestational week 18. To increase the enrollment rate, information regarding the trial and invitation to participate were also broadcasted online (Facebook, Instagram, and in an online magazine for pregnant women in Norway). Pregnant women who were interested in the study contacted the researchers at the Institute of Marine Research (IMR), Bergen, Norway.

Inclusion criteria were primiparous singleton pregnancy, gestational week ≤19, and Norwegian speaking and/or understand Norwegian writing. Exclusion criteria were allergies to fish and chronic diseases known to affect iodine status (Graves' disease, thyroiditis, thyroid nodules, known hypothyroidism or hyperthyroidism). Participants gave written informed consent after receiving written and oral information about the study. No specific information about iodine was given as the overall aim of the study was to study the relationship between Mommy's Food (nutrient intake in pregnancy and the infants' first year) and infant development. The women could withdraw from the study at any time without giving any reason. The trial complies with the Declaration of Helsinki and was approved by the Regional Committee for Medical and Health Research Ethics West (2015/879). The study protocol has been published elsewhere (18).

### Randomization and masking

Baseline data were collected at the first visit, and during gestational week 18–19. At the second visit in gestational week 19, the participants were randomized individually by lottery in blocks of 10 to ensure approximately equal allocation to both groups. Owing to the nature of the intervention, blinding of the participating mothers was not possible. Study investigators (L.K.M. and I.N.) generated the random allocation sequence, enrolled participants, and assigned participants to groups. Laboratory personnel were blinded when analyzing data with no access to the code. Study investigators (M.W.M. and S.N.) were blinded when analyzing the primary and secondary outcomes as the code was masked with dummy variables by a third study investigator (L.K.M.).

### Intervention

After randomization, participants in the intervention group received frozen cod fillets (Lerøy A/S, Bergen, Norway) and were instructed to consume two intervention meals of 200 g weekly (a total of 400 g cod per week) for 16 weeks (a total of 32 meals) from gestational week 20 to 36. The participants also received cod for their partner, if any, with the intention to increase compliance. The participants prepared the meals themselves and could choose their own recipes but were also provided with recipes that could be used *ad libitum*. For compliance purposes, participants were instructed to weigh (Kitchen Scale, Article No. 34–1207-16, ClasOhlson.com) the cod fillet before preparing the meals, and weigh the fillet leftovers (if any), after the meals were eaten. The participants recorded these data in a weight registration form, in addition to recipes used, and date of consumption.

To calculate total gram cod eaten each meal, gram cod after preparation of the meal was subtracted from gram cod before preparing the meal. Total gram cod for each meal during the intervention period was then summarized to get a value of total gram cod eaten during the intervention period. Total intake of cod was divided by 16 weeks to get a mean weekly intake of cod. Total intake of cod was divided by a maximum intake of cod during the intervention period (200 g × 32 meals = 6400 g) and multiplied with 100 to get a compliance score. Example of a total intake of 6400 g during the intervention or a mean intake of 400 g cod per week provided a compliance score of 100. The participants in the control group were instructed to continue to follow their habitual diet, without any restrictions.

### Outcomes

The primary outcome was UIC measured at postintervention. At the first visit in gestational week 18, participants received six marked collection tubes for the collection of urine samples on six consecutive days from gestational week 18–19, with instructions on how to collect the spot urine samples. Before the visit in gestational week 36, postintervention, participants received by mail six marked collection tubes for the collection of urine samples on six consecutive days from gestational week 35–36. The participants kept the urine samples in their home freezer until the visits in gestational week 19 and 36, respectively. Equal amounts of urine from the six spot urine samples (collected on six consecutive days between 4 pm and midnight) were homogenized into one pooled sample of 1 mL urine and were stored at −20°C in cryotubes (CryoTube™ Vials Nunc; Thermo Fischer Scientific, Roskilde, Denmark) pending analysis by inductively coupled plasma mass spectrometry (ICP-MS).

Before the analysis, the urine samples were defrosted in a refrigerator, diluted with 1% tetramethylammonium hydroxide filtrated using a sterile membrane filter (0.45 μm pore size), and transferred to tubes appropriate for the analysis by the Agilent 7500 for ICP-MS at IMR. Samples were analyzed against a urine calibration curve (standard addition curve) to measure the unknown iodine concentration (^127^I) in the collected urine samples. Accuracy was verified with certified reference material; Seronorm Trace Elements Urine (Nycomed Pharma, Norway), iodine content: 84 μg/L (range 72–96 μg/L) and 304 μg/L (range 260–348 μg/L). The measurement uncertainty of the method has been assessed based on internal reproducibility, analysis of standard reference material, and is set at 20% in the entire range (2–297 μg/L). The limit of detection was 2 μg/L.

For thyroid function and docosahexaenoic acid (DHA) analysis, blood samples were drawn in gestational week 18 and 36. Blood samples for serum preparation were collected in BD Vacutainer^®^ SST™ vials II *Advanced* and set to coagulate for a minimum of 30 minutes before centrifuging (1000–3000 *g*, room temperature, 10 minutes) within 60 minutes after extraction. Blood samples for red blood cell preparation were collected in BD Vacutainer K2E 5.4 mg vials and centrifuged (1000–1300 *g*, 20°C, 10 minutes) within 30 minutes.

Postseparation, serum samples were stored at −80°C pending analysis at Fürst Medical Laboratories (Norway), and red blood cell samples were stored at −80°C pending analysis at IMR. The serum samples were stored for a maximum of three months before analysis. Thyrotropin (TSH), free thyroxine (fT4), and free triiodothyronine (fT3) were analyzed in serum using magnetic separation and detection by chemiluminescence, labeled with acridinium ester, on an Advia Centaur XPT Immunoassay system (Siemens Healthcare Diagnostics, Inc., Tarrytown, NY). For all blood constitutes, the coefficient of variation was <6%. DHA was analyzed in red blood cells by a standardized procedures at IMR (19), using ultrafast gas chromatography (Thermo Electron Corporation, Franklin, MA).

The secondary outcome was neurodevelopment assessed by the cognitive, language, and motor scales of the *Bayley Scales of Infant and Toddler Developmental* third edition (Bayley-III) when the infants were 11 months of age. The Bayley-III is a comprehensive assessment tool of neurodevelopment administered directly with the child (20). The tool takes ∼45 to 60 minutes to administer and includes three main subscales: the cognitive, language (receptive and expressive), and motor (fine and gross motor) scales. The Bayley-III represents the gold standard of developmental assessment in this age group and is widely used as an outcome measure in clinical trials. The official Norwegian version of the Bayley-III translated and adapted for a Norwegian setting was used, with American norms from a representative American sample.

The Bayley-III provides 5 scaled scores [mean 10, range 1–19, standard deviation (SD) 3] and 3 composite scores (mean 100, range 40–160, SD 15) (21). Two trained testers (L.K.M. and I.N.), supervised by a neuropsychologist (M.H.) and a clinical child psychologist (I.K.), administered the Bayley-III in the current trial. Standardization exercises were conducted before the start of the study assessment until a satisfactory level of agreement was reached. During the trial, 20% of the tests were double scored with an interclass correlation ranging from 0.88 to 0.99, indicating high inter-rater agreement.

### Background variables

Demographic information, including education, income, prepregnancy and current weight, height, and nicotine use in pregnancy, was collected through an electronic questionnaire at baseline in gestational week 18–19. Gestational length and birthweight were obtained from birth records by the mother's recall. Preterm delivery was defined as birth <gestational week 37 and low birthweight as <2500 g.

### Iodine intake

Iodine intake was estimated from a structured six-day iodine-specific food diary designed and validated for this study (22). The food diary was filled out on six consecutive days at baseline and postintervention (between gestational week 18–19 and gestational week 35–36, respectively) at the exact same days as the spot urinary samples. The food diary included food items of iodine-rich foods (fish and seafood, milk and dairy products, and eggs) in addition to supplements used. The food diary was developed specifically for this study and has been validated and described in detail by Næss *et al.* (22). The article includes information of iodine content of the specific food items used, including cod, for calculation of iodine intake. For data on the use of iodine supplements since becoming pregnant, we used data from an iodine-specific food frequency questionnaire, which is also validated and described in detailed by Næss *et al.* (22). To retrieve the iodine content of the cod given in the intervention, 30 individual cod samples from the batch used in the intervention were analyzed at the IMR using ICP-MS, the method has previously been described in detail elsewhere (23).

### Statistical analysis

The power calculation for the sample size in the current study was based on data on a median (IQR) UIC of ∼80 (80) μg/L in pregnant women from the “Little in Norway” cohort (9,24). The sample size was calculated to detect a 30% difference in UIC between the intervention and control groups. The intended sample size was thus 60 individuals per group (total of 120) (0.05 one-tailed alpha, power 0.952). To account for attrition, 24 (20%) subjects were added reaching a final intended sample size of 144 (18,25).

The continuous variables are summarized as mean ± SD or median [interquartile range (IQR)], and the categorical variables are described in frequency and percent. Analyses were performed on an intention-to-treat basis, and missing cases were omitted from the data set (listwise deletion) (26). Normality was assessed by testing the distribution of continuous variables against a normal distribution using the Shapiro–Wilk *W* test. For the primary outcome, we present the median (IQR) UIC μg/L at baseline and postintervention. UIC data were transformed using log_10_ to correct for positive skewness.

In the main primary analysis, the Student's *t*-test was used to compare potential within- and between-group differences, and a one-way analysis of covariance (ANCOVA) was used to compare the differences in postintervention UIC μg/L between the groups, with baseline UIC μg/L included as a covariate. We included baseline UIC in the model as it is believed that the postintervention UIC to some degree depends on the baseline UIC μg/L. We also assessed whether UIC had remained stable, increased, or decreased between the baseline and postintervention. An increase and decrease were defined as a change larger than ±10% from baseline to postintervention, which were chosen according to the measurement uncertainty of 20%.

The Student's *t*-test was used to compare potential within- and between-group differences in thyroid hormone levels (TSH, fT3, and fT4), and a one-way ANCOVA was used to compare the differences in postintervention concentrations between the groups with the baseline concentrations included as a covariate.

For the secondary outcome, we present the mean (SD) of the Bayley-III cognitive composite score, the language scaled scores (receptive and expressive) and composite scores, and the motor scaled scores (fine and gross) and composite scores. We used a Student's *t*-test to compare the means between the groups. For all analyses, a two-sided *p*-value of < 0.05 was considered statistically significant. Statistical analyses were performed using Statistical Package for the Social Sciences (SPSS^®^ Statistics Version 25).

## Results

A total of 165 pregnant women showed interest in participating in the study by contacting the study secretariat. From January 2016 until February 2017, a total of 137 pregnant women were enrolled in the study and signed the informed consent. Between the enrollment and randomization, 4 participants withdrew from the study, and in total, 133 pregnant women were randomized to the intervention group (*n* = 68) or the control group (*n* = 65). Between randomization and post-testing, nine participants dropped out of the study [six (8.8%) in the intervention group and three (4.6%) in the control group]. In addition, two participants were lost to follow-up due to preterm delivery. Hence, for the primary outcome (UIC), 122 participants [*n* = 61 (89.7%) in the intervention group and *n* = 61 (93.8%) in the control group] were included in analysis.

**FIG. 1.**
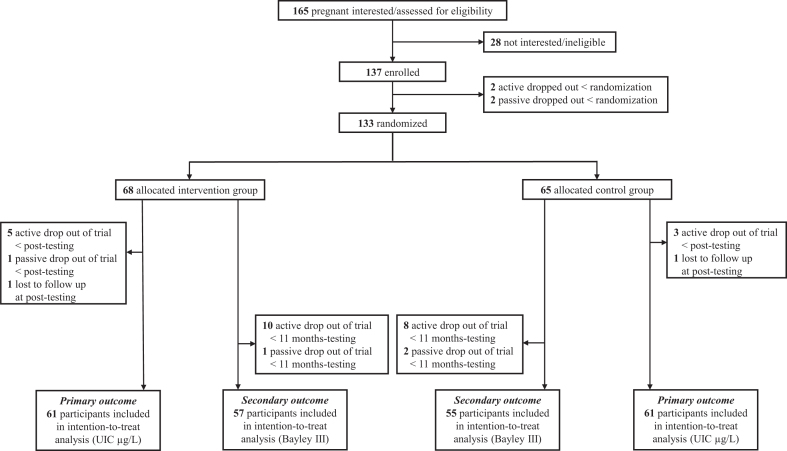
Trial profile depicting the flow of participants through the intervention trial. Bayley III, *Bayley Scales of Infant and Toddler Developmental* third edition; UIC, urinary iodine concentration.

For the secondary outcome (Bayley-III), 112 participants [*n* = 57 (83.8%) in the intervention group and *n* = 55 (84.6%) in the control group] were included in the analyses. There were no differences in any baseline characteristics between participants who withdrew from the study and those who completed the study at 11 months (Supplementary Table S1). An overview of the recruitment and flow of participants through the trial is outlined in Figure 1. Baseline characteristics for the randomized pregnant women were similar in the two groups (Table 1).

**Table 1.** Baseline Characteristics of Study Participants Within Each Group in the Mommy's Food Trial

Characteristic	Control		Intervention	
	*n*		*n*	
Age, years, mean (SD)	65	29.1 (3.5)	68	29.6 (4.0)
BMI, kg/m^2^	62	23.3 (4.3)	68	22.9 (3.9)
Education, years, %	63		68	
≥12	7	11.1	11	16.2
13–16	15	23.8	18	26.5
>16	41	65.1	39	57.4
Household income (NOK^a^), %	63		68	
Low (<200,000–549,000)	15	23.8	23	33.8
Medium (550,000–1,249,999)	40	63.5	36	52.9
High (1,250,000 to >2,000,000)	8	12.7	9	13.2
Nicotine use in pregnancy,^b^ yes, %	62		68	
≤gestational week 8	7	11.3	5	7.4
>gestational week 8	0	0	0	0
Iodine intake, μg/day, median (IQR)^c^	65	133 (81–240)	68	152 (92–267)
Milk and dairy	65	67 (43–92)	68	62 (22–76)
Egg	65	7 (3–14)	68	7 (3–14)
Seafood^b^	65	33 (94)	68	42 (94)
Supplements (users only)	21	175 (135–200)	25	150 (131–175)
Iodine supplement use since becoming pregnant, *n* (%)^d^	59	21 (32)	63	29 (43)

^a^ One hundred NOK = ∼11.6 USD/10.2 EUR.

^b^ No participants reported use of nicotine after gestational week 8.

^c^ Intake estimated from six-day food dairy at baseline.

^d^ Reported from food frequency questionnaire.

BMI, body mass index; IQR, interquartile range; NOK, Norwegian Krone; SD, standard deviation.

Mean compliance score was 77, ranging from 35 to 102. More than 70% of the participants had a compliance score of more than 70. Approximately 50% of the participants had a compliance score of more than 80. Less than 10% had a compliance score of <50. The mean (SD), median (IQR), and 5 percentile intakes of the received cod in the intervention group were 306 (62), 318 (275–356), and 175 g per week, respectively. The mean (IQR, min, max) analyzed iodine content of the cod given in the intervention based on analysis of 30 individual cod samples was 81 (46–71, 31, 630) μg/100 g. There were no reported adverse events during or after the intervention.

In total, six infants were delivered preterm (<gestational week 37) (*n* = 3 in the control group, *n* = 3 in the intervention group), and three infants were born with low birthweight (<2500 g) (*n* = 2 in the control group, *n* = 1 in the intervention group). There was no difference between gestational length [control group: mean (SD): 40.2 (2.6) weeks, intervention group: mean (SD): 40.3 (1.8) weeks] and birthweight [control group: mean (SD): 3442 (589) g, intervention group: mean (SD): 3541 (478) g] between the groups.

For the main analysis, the UIC was significantly higher in the intervention group postintervention [median (IQR) 98 (64–145) μg/L], compared with the control group [median (IQR) 73 (52–120) μg/L] (*p* = 0.028). The difference between the groups was still significant after adjusting for baseline UIC μg/L [log mean (SD) control group: 1.89 (0.027), log mean (SD) intervention group: 1.96 (0.027), mean difference between log means: 0.076 [confidence interval, CI 0.001–0.150], *p* = 0.048] (Table 2). The estimated iodine intake postintervention was significantly higher (*p* = 0.001) in the intervention group [median (IQR, min, max) 218 (156–323, 45, 481) μg/day, *n* = 61] compared with the control group [median (IQR, min, max) 146 (87–264, 32, 423) μg/day, *n* = 61].

**Table 2.** Urinary Iodine Concentration at Baseline and Postintervention in the Mommy's Food Trial Participants

	UIC, μg/L^a^		Difference between groups postintervention	
	Baseline, median (IQR)	Post, median (IQR)	Crude, *p*^b^	Adjusted, *p*^c^
Control (*n* = 61)	85 (55–130)	73 (52–120)	*0*.*028*	*0*.*048*^d^
Intervention (*n* = 61)	88 (64–130)	98 (64–145)		

^a^ Analyzed in a pooled sample of six spot samples collected on six consecutive days at each time point.

^b^ Independent sampled *t*-test for comparison of log-transformed values.

^c^ One-way ANCOVA for comparison of differences between control and intervention groups adjusted for baseline UIC (μg/L). A two-sided *p*-value of < 0.05 was considered statistically significant.

^d^ η^2^ = 0.033.

ANCOVA, analysis of covariance; UIC, urinary iodine concentration.

For the secondary outcome, the intervention group had a significant lower cognitive composite score on the Bayley-III compared with the control group, when the infants were 11 months of age [log mean (SD) control group: 1.99 (0.049), log mean (SD) intervention group: 1.97 (0.04), mean difference between log means: 0.016 [CI 0.0004–0.0321], *p* = 0 · 045]. There were no significant differences in the Bayley-III language- or motor composite scores between the groups (Table 3).

**Table 3.** Infant Neurodevelopment Assessed at 11 Months of Age in the Mommy's Food Trial Participants

Neurodevelopment scores^a^	Control (*n* = 55), mean (SD)	Intervention (*n* = 57), mean (SD)	*p*^b^
Cognitive composite score	99 (10)	95 (9)	*0*.*045*^c^
Language composite scores	95 (8)	96 (8)	*0*.*67*
Receptive language-scaled score	8 (2)	8 (2)	*0*.*20*
Expressive language-scaled score	11 (1)	11 (1)	*0*.*36*
Motor composite scores	94 (8)	92 (7)	*0*.*24*
Gross motor-scaled score	9 (2)	9 (2)	*0*.*85*
Fine motor-scaled score	9 (1)	9 (2)	*0*.*13*

^a^ Neurodevelopment was assessed by the cognitive, language, and motor scales of the *Bayley Scales of Infant and Toddler Developmental* third edition.

^b^ Independent *t*-test of differences for comparison of log-transformed values between groups. Language composite scores owing to normality. A two-sided *p*-value of < 0.05 was considered statistically significant.

^c^ Cohen's *d* = 0.42.

Table 4 shows change in UIC from baseline to postintervention in the control and intervention groups. The number of participants with an increase in UIC was 28% in the control group and 36% in the intervention group, while the number of participants with a decrease in UIC was 51% and 36% in the control and intervention groups, respectively. The thyroid hormones TSH, fT3, and fT4 did not differ between the groups postintervention (Table 5). There was no difference in DHA status neither at baseline nor postintervention between the groups (Supplementary Table S2).

**Table 4.** Change in Urinary Iodine Concentration from Baseline to Postintervention in the Mommy's Food Trial Participants

UIC baseline, μg/L	UIC postintervention						*p*^c^
	Control			Intervention			
	Increase,* n *(%)^a^	Decrease,* n *(%)^a^	Stable,* n *(%)^a^	Increase,* n *(%)^b^	Decrease,* n *(%)^b^	Stable,* n *(%)^b^	
<50	4 (7)	5 (8)	3 (5)	7 (12)	0	0	*0*.*034*
50–100	9 (15)	9 (15)	4 (7)	15 (25)	7 (12)	9 (15)	*0*.*243*
>100	4 (7)	17 (28)	6 (10)	3 (5)	15 (25)	5 (8)	*0*.*847*
All	17 (28)	31 (51)	13 (21)	25 (41)	22 (36)	14 (23)	*0*.*081*

Increase and decrease were defined as a change larger than ±10% from baseline to postintervention.

^a^ Percent within control group.

^b^ Percent within intervention group.

^c^ Differences tested between increase and decrease in the control and intervention groups using Pearson's chi-square test or Fisher's exact test. A two-sided *p*-value of < 0.05 was considered statistically significant.

**Table 5.** Thyrotropin, Free Triiodothyronine, and Free Thyroxine in Intervention and Control Groups at Baseline (gw 18) and Postintervention (gw 36) in the Mommy's Food Trial Participants

	Thyroid hormones		Difference between groups postintervention	
	Baseline, mean (SD)	Post, mean (SD)	Crude, p^a^	Adjusted, p^b^
TSH, mIU/L				
Control (*n* = 58)	1.6 (0.7)	1.8 (0.8)	*0*.*94*	*0*.*89*
Intervention (*n* = 61)	1.6 (0.8)	1.8 (0.8)		
fT3, pmol/L				
Control (*n* = 58)	4.3 (0.4)	3.8 (0.4)	*0*.*18*	*0*.*10*
Intervention (*n* = 61)	4.2 (0.5)	3.9 (0.4)		
fT4, pmol/L				
Control (*n* = 58)	13.8 (1.5)	13.5 (1.9)	*0*.*87*	*0*.*60*
Intervention (*n* = 61)	14.0 (1.7)	13.4 (1.4)		

^a^ Independent *t*-test of differences between groups postintervention.

^b^ One-way ANCOVA for comparison of differences between control and intervention groups adjusted for baseline levels. A two-sided *p*-value of < 0.05 was considered statistically significant.

fT3, free triiodothyronine; fT4, free thyroxine; gw, gestational week; TSH, thyrotropin.

## Discussion

To our knowledge, this is the first RCT to investigate the effect of nonfortified iodine-rich food consumption in pregnancy on maternal iodine status and infant neurodevelopment. In concordance with other recent studies in Norwegian and other European pregnant populations, the pregnant women in this study were mildly-to-moderately iodine deficient at baseline. After the intervention with two meals of cod for a period of 16 weeks in pregnancy (gestational week 20–36), median UIC was significantly higher in the intervention group compared with the control group. However, the median UIC values remained below the recommended UIC of 150 μg/L. The children's language and motor scores on the Bayley-III were similar in both groups when assessed at 11 months of age, while the cognitive score was better in the control group.

Our results suggest that it is possible to improve the iodine status during pregnancy using a nutrition-sensitive food system approach to meet the dietary requirements of a population (27). Several RCTs have previously been conducted to improve the iodine status in pregnancy, using iodine-containing supplements (28). In countries without iodized salt, such as in Norway and the United Kingdom, it is important to include one or several key sources of iodine in the habitual diet to achieve an adequate iodine intake. The present study shows that cod has a potential as an important dietary source of iodine in pregnancy when included in the diet. However, median UIC after the intervention did not meet the epidemiological criteria for adequate iodine nutrition (median UIC between 150 and 250 μg/L) suggested by the WHO (2). Although the suggested limit of ∼100 μg/L, based on the Nordic Nutrition Recommendations, was almost met with a median of 98 μg/L in the intervention group (5).

The intervention group had a significantly lower score on the cognitive composite score than the control group, while the language and motor scores were similar between the groups. Since this is the first RCT aiming at increasing iodine status in pregnancy through a food-based approach, and subsequently assessing child development, comparable studies are lacking. In regions of severe iodine deficiency, iodine supplementation in pregnancy has been shown to reduce the incidence of cretinism and improve motor development in children (16). However, the effects of iodine supplementation during pregnancy in mildly-to-moderately iodine-deficient populations are still unclear (16). A Cochrane review from 2017, including 11 trials, concluded that there was insufficient evidence for either the benefit or harm of iodine supplementation during pregnancy on child neurodevelopment due to small participant numbers and low quality of trials (28). Only two of the RCTs included developmental outcomes in children, and in those, there were no difference in the developmental outcomes between the groups (29,30).

The first randomized, double-blinded, placebo-controlled trial in mildly-to-moderately iodine-deficient pregnant women was conducted from 2008 to 2011 in India and Thailand. They found no effect of 200 μg iodine per day from early pregnancy (<gestational week 14) until delivery on child cognition at 5–6 years of age (31). It is important to note that although the median UIC of the two cohorts was indicative of mild deficiency at baseline, one of the cohorts was actually iodine sufficient, which potentially could have confounded the results. However, an analysis of secondary outcomes at age one year showed higher scores for expressive language measured by the Bayley III in the placebo group than in the iodine intervention group (31). Likewise, we found a higher Bayley III score on cognition in the control group than in the intervention group. However, the effect size of the difference between the groups was small, and the mean cognitive level was close to average for both the control and intervention groups. Still, the mean difference in Bayley-III cognitive score of four points could reflect important differences in learning and development. Follow-up studies investigating stability and trajectories are needed to conclude on the impact of these differences. Although more uncertain on an individual level, small effects may be meaningful on a public health level.

Results from a few RCTs and observational studies suggest that introducing an iodine supplement after the onset of pregnancy in women with mild-to-moderate iodine deficiency can have adverse effects on thyroid hormones and infant development (28,32–35). Furthermore, a recent systematic review and meta-analysis of the effects of iodine supplementation on thyroid function and child neurodevelopment in mildly-to-moderately iodine-deficient pregnant women concluded that there is insufficient good-quality evidence to support current recommendations for iodine supplementation in pregnancy in areas of mild-to-moderate deficiency (36). Despite these ambiguous results, routine iodine supplementation in pregnancy is recommended by health authorities across the world.

The possible underlying mechanisms of all iodine deficiency disorders (IDDs), including impaired child development, are inadequate thyroid hormone production and their disturbed action in target tissues (37). Correcting iodine deficiency has proven beneficial to prevent IDDs worldwide (38,39). However, iodine supplementation or fortification has also been associated with increased incidence of thyroid dysfunction (40). Even though we managed to increase the iodine status in the intervention group, it might have been too late as the first trimester of pregnancy is crucial as the fetal brain development is dependent on maternal thyroid hormone transfer (41). Moreover, not all participants in the intervention group had an increase in UIC from baseline to postintervention, which may have interfered with the secondary outcome. Still, for those participants who had a UIC <50 μg/L at baseline, the intervention group differed from the control group in that all the participants had an increase in UIC from baseline to postintervention. Nevertheless, we cannot exclude the possibility that the alterations in iodine intake during pregnancy could have a negative effect on maternal thyroid function and hence child cognition. Noteworthy, a temporal stunning of thyroid function has been discussed to be as a consequence of changes in iodine intake in mildly-to-moderately iodine-deficient populations, and not solely as a consequence of excessive iodine intake (42,43). In the current study, the women's iodine intake could not be characterized as excessive.

The differences in UIC concentration and estimated iodine intake between groups after the intervention were not reflected in the thyroid hormones that were similar in the groups postintervention. However, individuals have a genetic set point for thyroid hormone concentrations and, despite a wide interindividual variation, there is a low index of individuality (44). Nonetheless, there is a possibility that a small change in the habitual maternal iodine intake during pregnancy, rather than before pregnancy, might have changed fetal thyroid hormone status during this vulnerable period of neurodevelopment. Thus, our finding urges further attention.

A key limitation in this study could be the onset of the intervention as timing of exposure to maternal iodine insufficiency is a modulator for its effect on outcomes (45). In the attempt of a nonbiased source population, the women were recruited before their first meeting with the public health care system at gestational week 18. Thus, baseline testing, randomization, and onset of the dietary intervention did not commence before gestational week 18 and 19. Blinding is a challenge in RCTs with food. We have previously conducted RCTs with fish in kindergarten children and youths preparing identical meals to intervention and control groups, the only difference being the protein source (46,47). However, due to both texture and taste, blinding was not possible. RCTs with single dietary components are inconclusive in confirming health outcomes from observational studies. This could be due to the complexity of food containing several bioactive components. Thus, RCTs with food are still valuable despite the challenge of blinding (48).

A limitation or rather a consequence of a dietary intervention introducing a food (e.g., lean fish) twice a week is replacement of another habitual food (e.g., fatty fish). While lean fish is a good source of iodine, fatty fish is an excellent source of DHA, a nutrient also playing a critical role in brain development (49,50). Details regarding the seafood intake of the participants in this study have recently been described in detail elsewhere (51). While the control group had a stable intake of both lean and fatty fish, the intervention group increased its intake of lean fish and decreased the intake of fatty fish from baseline to postintervention. The intervention group had a higher DHA status compared with the control group postintervention, although the difference was not significant. Still, the higher DHA status in the intervention group is not likely to confound the effects of interest or have a large impact on the cognitive outcomes.

As lean fish also is a source of mercury, we also measured mercury status at baseline and postintervention (51). However, the concentrations of mercury were low and only a small difference between the groups was observed; so, we do not believe this has affected the results. Furthermore, the iodine content of the cod given in the intervention was low compared with the reported values in the Norwegian food composition table. Laboratory analysis also showed large variation in the individual cod fillets. Thus, the dose given in each meal and to each participant in the intervention group is believed to have varied, introducing a dose bias.

We did not correct for multiple testing for the secondary outcome, and thus, the risk of detecting false-positive results (type 1 error) might have increased. However, correction for multiple testing has been debated as it also decreases the statistical power and increases the risk of not detecting real differences (type 2 error) (52).

Strengths of this study are the high compliance and the analysis of a pooled sample of six spot urine samples at baseline and postintervention in both groups, including both weekdays and weekend. Although 10 samples are suggested for assessing individual iodine status, 6 samples could be enough to categorize individual status based on UIC as there was a strong agreement with the food diary, recorded on the same six consecutive days (22,53).

In conclusion, an increased intake of iodine through consumption of cod in mildly-to-moderately iodine-deficient pregnant women resulted in an increased iodine status measured as UIC. The intervention had no measurable effect on maternal thyroid function, and the infants of the mothers in the intervention group had a lower cognitive score compared with the control group. A follow-up of these children over time will indicate if these differences persist. While the overall literature is ambiguous, there is a need to investigate further both positive and possible adverse effects of increasing iodine intake in pregnancy, rather than ideally before conception, to assure the safety of the present guidelines.

## Supplementary Material

Supplemental data

Supplemental data

## Availability of Data Materials

Requests for data collected in the Mommy's Food study (such as deidentified participant data) can be made to the corresponding author following publication, and requests will be considered on an individual basis. Any requests require completion and approval of the application for use of data from the Mommy's Food study. The trial project group will review and, if acceptable and approved by the Regional Committee for Medical and Health Research Ethics West, Norway, provide approval of the request. A signed data sharing access agreement will be required. The data will be provided as an SPSS data set. Any other format requests might incur costs to the requestor. To facilitate the data access process, please contact, data will be available after publication of the study results, Maria Wik Markhus at maria.wik.markhus@hi.no and mammasmat@hi.no.

## References

[1] Andersson M, Karumbunathan V, Zimmermann MB 2012 Global iodine status in 2011 and trends over the past decade. J Nutr 142:744–750 22378324 10.3945/jn.111.149393

[2] WHO, UNICEF, ICCIDD. 2007 Assessment of Iodine Deficiency Disorders and Monitoring Their Elimination. World Health Organisation, Geneva, Switzerland

[3] Zimmermann MB, Gizak M, Abbott K, Andersson M, Lazarus JH 2015 Iodine deficiency in pregnant women in Europe. Lancet Diabetes Endocrinol 3:672–674 26268907 10.1016/S2213-8587(15)00263-6

[4] Zimmermann MB 2008 Iodine deficiency in pregnancy and the effects of maternal iodine supplementation on the offspring: a review. Am J Clin Nutr 89:668S–672S 19088150 10.3945/ajcn.2008.26811C

[5] Nordic Council of Ministers 2014 Nordic Nutrition Recommendations 2012: Integrating Nutrition and Physical Activity. Nordic Council of Ministers, Copenhagen, Denmark

[6] Abel MH, Korevaar TI, Erlund I, Villanger GD, Caspersen IH, Arohonka P, Alexander J, Meltzer HM, Brantsæter AL 2018 Iodine intake is associated with thyroid function in mild to moderately iodine deficient pregnant women. Thyroid 28:1359–1371 30132420 10.1089/thy.2018.0305 PMC6157349

[7] Markhus M, Dahl L, Moe V, Abel M, Brantsæter A, Øyen J, Meltzer H, Stormark K, Graff I, Smith L 2018 Maternal iodine status is associated with offspring language skills in infancy and toddlerhood. Nutrients 10:1270 10.3390/nu10091270 PMC6163597 30205599

[8] Brantsæter AL, Abel M, Haugen M, Meltzer HM 2013 Risk of suboptimal iodine intake in pregnant Norwegian women. Nutrients 5:424–440 23389302 10.3390/nu5020424 PMC3635203

[9] Dahl L, Markhus MW, Sanchez P, Moe V, Smith L, Meltzer HM, Kjellevold M 2018 Iodine deficiency in a study population of Norwegian pregnant women—results from the little in Norway Study (LiN). Nutrients 10:513 10.3390/nu10040513 PMC5946298 29677112

[10] Henjum S, Aakre I, Lilleengen A, Garnweidner-Holme L, Borthne S, Pajalic Z, Blix E, Gjengedal E, Brantsæter AL 2018 Suboptimal iodine status among pregnant women in the Oslo area, Norway. Nutrients 10:280 10.3390/nu10030280 PMC5872698 29495606

[11] Berg V, Nøst TH, Skeie G, Thomassen Y, Berlinger B, Veyhe AS, Jorde R, Odland JØ, Hansen S 2017 Thyroid homeostasis in mother–child pairs in relation to maternal iodine status: the MISA study. Eur J Clin Nutr 71:1002 28537582 10.1038/ejcn.2017.83 PMC5543254

[12] The Norwegian Directorate of Health 2019 Utviklingen i norsk kosthold 2018 [Trends in the Norwegian diet]. The Norwegian Directorate of Health, Oslo

[13] Carlsen M, Andersen L, Dahl L, Norberg N, Hjartåker A 2018 New iodine food composition database and updated calculations of iodine intake among Norwegians. Nutrients 10:930 10.3390/nu10070930 PMC6073680 30037013

[14] Pharoah P, Buttfield I, Hetzel B 1971 Neurological damage to the fetus resulting from severe iodine deficiency during pregnancy. Lancet 297:308–310 10.1016/s0140-6736(71)91040-3 4100150

[15] WHO and United Nations Children's Fund 2007 Reaching optimal iodine nutrition in pregnant and lactating women and young children. World Health Organization, Geneva, Switzerland 10.1017/s1368980007705360 18333291

[16] Zhou SJ, Anderson AJ, Gibson RA, Makrides M 2013 Effect of iodine supplementation in pregnancy on child development and other clinical outcomes: a systematic review of randomized controlled trials. Am J Clin Nutr 98:1241–1254 24025628 10.3945/ajcn.113.065854

[17] Pearce EN, Lazarus JH, Moreno-Reyes R, Zimmermann MB 2016 Consequences of iodine deficiency and excess in pregnant women: an overview of current knowns and unknowns. Am J Clin Nutr 104(Suppl 3):918S–923S 27534632 10.3945/ajcn.115.110429 PMC5004501

[18] Markhus MW, Kvestad I, Midtbø LK, Nerhus I, Ødegaard ER, Graff IE, Lie Ø, Dahl L, Hysing M, Kjellevold M 2018 Effects of cod intake in pregnancy on iodine nutrition and infant development: study protocol for Mommy's Food—a randomized controlled trial. BMC Nutr 4:7 32153871 10.1186/s40795-018-0215-1 PMC7050745

[19] Araujo P, Nguyen T-T, Frøyland L, Wang J, Kang JX 2008 Evaluation of a rapid method for the quantitative analysis of fatty acids in various matrices. J Chromatogr A 1212:106–113 18937951 10.1016/j.chroma.2008.10.006 PMC2593112

[20] Bayley N 2006 Bayley Scales of Infant and Toddler Development. PsychCorp, Pearson

[21] Bayley N 2009 Bayley Scales of Infant and Toddler Development (Bayley-III) Norsk Manual Supplement, 3rd ed., Pearson, San Antonio, TX

[22] Næss S, Aakre I, Kjellevold M, Dahl L, Nerhus I, Midtbø LK, Markhus MW 2019 Validation and reproducibility of a new iodine specific food frequency questionnaire for assessing iodine intake in Norwegian pregnant women. Nutr J 18:62 31665021 10.1186/s12937-019-0489-4 PMC6821006

[23] Nerhus I, Markhus MW, Nilsen BM, Øyen J, Maage A, Ødegård ER, Midtbø LK, Frantzen S, Kögel T, Graff IE 2018 Iodine content of six fish species, Norwegian dairy products and hen's egg. Food Nutr Res 62 DOI: 10.29219/fnr.v62.1291 PMC5971469 29853825

[24] Moe V, Fredriksen E, Kjellevold M, Dahl L, Markhus MW, Stormark KM, von Soest T, Olafsen KS, Vannebo UT, Smith L 2019 Little in Norway: a prospective longitudinal community-based cohort from pregnancy to child age 18 months. BMJ Open 9:e031050 10.1136/bmjopen-2019-031050 PMC6955541 31892648

[25] Faul F, Erdfelder E, Lang A-G, Buchner A 2007 G* Power 3: a flexible statistical power analysis program for the social, behavioral, and biomedical sciences. Behav Res Methods 39:175–191 17695343 10.3758/bf03193146

[26] Nich C, Carroll KM 2002 ‘Intention-to-treat'meets ‘missing data’: implications of alternate strategies for analyzing clinical trials data. Drug Alcohol Depend 68:121–130 12234641 10.1016/s0376-8716(02)00111-4 PMC3651592

[27] Uccello E, Kauffmann D, Calo M, Streissel M 2017 Nutrition-Sensitive Agriculture and Food Systems in Practice. FAO, Rome, Italy

[28] Harding KB, Peña-Rosas JP, Webster AC, Yap CM, Payne BA, Ota E, De-Regil LM 2017 Iodine supplementation for women during the preconception, pregnancy and postpartum period. Cochrane Database Syst Rev 3:CD011761 28260263 10.1002/14651858.CD011761.pub2 PMC6464647

[29] Brucker-Davis F, Ganier-Chauliac F, Gal J, Panaïa-Ferrari P, Pacini P, Fénichel P, Hiéronimus S 2015 Neurotoxicant exposure during pregnancy is a confounder for assessment of iodine supplementation on neurodevelopment outcome. Neurotoxicol Teratol 51:45–51 26247661 10.1016/j.ntt.2015.07.009

[30] Zhou SJ, Skeaff SA, Ryan P, Doyle LW, Anderson PJ, Kornman L, Mcphee AJ, Yelland LN, Makrides M 2015 The effect of iodine supplementation in pregnancy on early childhood neurodevelopment and clinical outcomes: results of an aborted randomised placebo-controlled trial. Trials 16:563 26654905 10.1186/s13063-015-1080-8 PMC4675066

[31] Gowachirapant S, Jaiswal N, Melse-Boonstra A, Galetti V, Stinca S, Mackenzie I, Thomas S, Thomas T, Winichagoon P, Srinivasan K, Zimmermann MB 2017 Effect of iodine supplementation in pregnant women on child neurodevelopment: a randomised, double-blind, placebo-controlled trial. Lancet Diabetes Endocrinol 5:853–863 29030199 10.1016/S2213-8587(17)30332-7

[32] Murcia M, Rebagliato M, Iniguez C, Lopez-Espinosa M-J, Estarlich M, Plaza B, Barona-Vilar C, Espada M, Vioque J, Ballester F 2011 Effect of iodine supplementation during pregnancy on infant neurodevelopment at 1 year of age. Am J Epidemiol 173:804–812 21385833 10.1093/aje/kwq424

[33] Rebagliato M, Murcia M, Álvarez-Pedrerol M, Espada M, Fernández-Somoano A, Lertxundi N, Navarrete-Muñoz E-M, Forns J, Aranbarri A, Llop S, Julvez J, Tardón A, Ballester F 2013 Iodine supplementation during pregnancy and infant neuropsychological development: INMA Mother and Child Cohort Study. Am J Epidemiol 177:944–953 23548753 10.1093/aje/kws333

[34] Abel MH, Caspersen IH, Meltzer HM, Haugen M, Brandlistuen RE, Aase H, Alexander J, Torheim LE, Brantsaeter AL 2017 Suboptimal maternal iodine intake is associated with impaired child neurodevelopment at 3 years of age in the Norwegian Mother and Child Cohort Study. J Nutr 147:1314–1324 28515161 10.3945/jn.117.250456

[35] Abel MH, Brandlistuen RE, Caspersen IH, Aase H, Torheim LE, Meltzer HM, Brantsaeter AL 2018 Language delay and poorer school performance in children of mothers with inadequate iodine intake in pregnancy: results from follow-up at 8 years in the Norwegian Mother and Child Cohort Study. Eur J Nutr 58:3047–3058 30417257 10.1007/s00394-018-1850-7 PMC6842354

[36] Dineva M, Fishpool H, Rayman MP, Mendis J, Bath SC 2020 Systematic review and meta-analysis of the effects of iodine supplementation on thyroid function and child neurodevelopment in mildly-to-moderately iodine-deficient pregnant women. Am J Clin Nutr 112:389–412 32320029 10.1093/ajcn/nqaa071

[37] Eastman CJ, Zimmermann MB 2018 The iodine deficiency disorders Endotext [Internet]. MDText.com, Inc., South Dartmouth, MA

[38] Pearce EN, Andersson M, Zimmermann MB 2013 Global iodine nutrition: where do we stand in 2013? Thyroid 23:523–528 23472655 10.1089/thy.2013.0128

[39] Markou K, Georgopoulos N, Kyriazopoulou V, Vagenakis A 2001 Iodine-induced hypothyroidism. Thyroid 11:501–510 11396709 10.1089/105072501300176462

[40] Laurberg P, Jørgensen T, Perrild H, Ovesen L, Knudsen N, Pedersen IBl, Rasmussen LB, Carlé A, Vejbjerg P 2006 The Danish investigation on iodine intake and thyroid disease, DanThyr: status and perspectives. Eur J Endocrinol 155:219–228 16868134 10.1530/eje.1.02210

[41] Bernal J, Nunez JJ 1995 Thyroid hormones and brain development. Eur J Endocrinol 133:390–398 7581959 10.1530/eje.0.1330390

[42] Zimmermann MB 2008 Iodine requirements and the risks and benefits of correcting iodine deficiency in populations. J Trace Elem Med Biol 22:81–92 18565420 10.1016/j.jtemb.2008.03.001

[43] Delange F 1994 The disorders induced by iodine deficiency. Thyroid 4:107–128 8054857 10.1089/thy.1994.4.107

[44] Waise A, Price HC 2009 The upper limit of the reference range for thyroid-stimulating hormone should not be confused with a cut-off to define subclinical hypothyroidism. Ann Clin Biochem 46:93–98 19164339 10.1258/acb.2008.008113

[45] Zoeller R, Rovet J 2004 Timing of thyroid hormone action in the developing brain: clinical observations and experimental findings. J Neuroendocrinol 16:809–818 15500540 10.1111/j.1365-2826.2004.01243.x

[46] Øyen J, Kvestad I, Midtbø LK, Graff IE, Hysing M, Stormark KM, Markhus MW, Baste V, Frøyland L, Koletzko B 2018 Fatty fish intake and cognitive function: FINS-KIDS, a randomized controlled trial in preschool children. BMC Med 16:41 29530020 10.1186/s12916-018-1020-z PMC5848440

[47] Handeland K, Skotheim S, Baste V, Graff IE, Frøyland L, Lie Ø, Kjellevold M, Markhus MW, Stormark KM, Øyen J 2018 The effects of fatty fish intake on adolescents' nutritional status and associations with attention performance: results from the FINS-TEENS randomized controlled trial. Nutr J 17:30 29475446 10.1186/s12937-018-0328-z PMC5824444

[48] Jacobs DR, Tapsell LC 2007 Food, not nutrients, is the fundamental unit in nutrition. Nutr Rev 65:439–450 17972438 10.1111/j.1753-4887.2007.tb00269.x

[49] Lauritzen L, Brambilla P, Mazzocchi A, Harsløf L, Ciappolino V, Agostoni C 2016 DHA effects in brain development and function. Nutrients 8:6 10.3390/nu8010006 PMC4728620 26742060

[50] Innis SM 2007 Dietary (n-3) fatty acids and brain development. J Nutr 137:855–859 17374644 10.1093/jn/137.4.855

[51] Næss S, Kjellevold M, Dahl L, Nerhus I, Midtbø LK, Bank MS, Rasinger JD, Markhus MW 2020 Effects of seafood consumption on mercury exposure in Norwegian pregnant women: a randomized controlled trial. Environ Int 141:105759 32388274 10.1016/j.envint.2020.105759

[52] Rothman KJ 2014 Six persistent research misconceptions. J Gen Intern Med 29:1060–1064 24452418 10.1007/s11606-013-2755-z PMC4061362

[53] König F, Andersson M, Hotz K, Aeberli I, Zimmermann MB 2011 Ten repeat collections for urinary iodine from spot samples or 24-hour samples are needed to reliably estimate individual iodine status in women. J Nutr 141:2049–2054 21918061 10.3945/jn.111.144071

