# Supplementary material for: Effects of Two Weekly Servings of Cod for 16 Weeks in Pregnancy on Maternal Iodine Status and Infant Neurodevelopment: Mommy's Food, a Randomized-Controlled Trial
Source: Thyroid. 2021 Feb 12;31(2):288–98. doi: 10.1089/thy.2020.0115 (PMC7891220; doi:10.1089/thy.2020.0115)
Supplement: Supplemental data [file Supp_TableS1.pdf]

Supplementary Table 1. Difference in baseline characteristics between completers 11 months and non-completers 11 months.

**Characteristic**	***n***	**Completers**	***n***	**Non-completers**	***p-value*[b]**
Age, in years, mean (SD)	114	29.4 (3.7)	21	28.5 (4.4)	0.50
BMI, kg/m^2	112	23.2 (4.2)	20	22.5 (3.0)	0.81
Education, %	112		68		
≥ 12 years	13	11.6	6	28.6	
13-16 years	30	26.8	3	14.3	
> 16 years	69	61.6	12	57.1	
Household Income (NOK[a]), %	112		21		0.38
Low (<200 000 - 549 999)	31	27.2	8	34.8	
Medium (550 000 - 1 249 999)	65	57.0	12	52.2	
High (1 250 000) > 2 000 000)	16	14.0	1	4.3	
Nicotine use in pregnancy c, yes, %	112		20		
≤ gestational week 8	11	9.8	1	5.0	NA
> gestational week 8	0		0	0	
Iodine intake (µg/day), median (IQR)	114	152 (87-255)	20	143 (88-281)	0.83

[a] One hundred NOK=*11.6 USD/10.2 EUR.

[b] Paired sampled *t*-test for comparison of numerical values and Pearson's chi square test for comparison of categorical values between groups.

[c] No participants reported use of nicotine after gestational week 8.

Abbreviations: SD. Standard deviation; BMI, body mass index; NOK, Norwegian Krone; IQR, inter quartile range.
