# Supplementary material for: Effects of Two Weekly Servings of Cod for 16 Weeks in Pregnancy on Maternal Iodine Status and Infant Neurodevelopment: Mommy's Food, a Randomized-Controlled Trial
Source: Thyroid. 2021 Feb 12;31(2):288–98. doi: 10.1089/thy.2020.0115 (PMC7891220; doi:10.1089/thy.2020.0115)
Supplement: Supplemental data [file Supp_TableS2.pdf]

Supplementary Table 2. Red blood cell DHA concentration at baseline and post intervention in the Mommy's Food trial participants.

	**RBC DHA (μg/g)**		**Difference between groups post intervention**	
	Baseline	Post	Crude	Adjusted
	Mean (SD)	Mean (SD)	*p*-value [a]	*p*-value [b]
**Control (*n*= 55)**	136 (24)	137 (41)	*0.13*	*0.09*
**Intervention (*n*= 61)**	140 (37)	149 (39)		

[a] Independent sampled t-test for comparison of log transformed values. [b] One-way analysis of covariance (ANCOVA) for comparison of differences between control and intervention group adjusted for pre DHA (μg/g).
Abbreviations: DHA, docosahexaenoic acid; RBC, red blood cell.
